# Supplementary material for: Improving the Activity of Tryptophan Synthetase via a Nucleic Acid Scaffold
Source: Molecules. 2023 Oct 26;28(21):7272. doi: 10.3390/molecules28217272 (PMC10648251; doi:10.3390/molecules28217272)
Supplement: Supplementary file 1 [file molecules-28-07272-s001.zip › molecules-2471801-supplementary.pdf]

dCE2
SKRNKPGKATGKGKPVGDKWLDDAGKDSGAPIPDRIADKLRDKEFKNFDD
FRKKFWEEVSKDPDLSKQFKGSNKTNIQKGKAPFARKKDQVGGRERFELN
HDKPISQDGGVYDMNNIRVTTPKRNIDIERGK

dCE7
SKRNKPGKATGKGKPVNNKWLNNAGKDLGSPVPDRIANKLRDKEFKSFDD
FRKKFWEEVSKDPELSKQFSRNNNDRMKVGKAPKTRTQDVSGKRTSFELN
HEKPISQNGGVYDMDNISVVTPKRNIDIERGK

dCE8
SKRNKPGKATGKGKPVGDKWLDDAGKDSGAPIPDRIADKLRDKEFKNFDD
FRRKFWEEVSKDPELSKQFNPGNKKRLSQGLAPRARNKDTVGGRRSFELN
HDKPISQDGGVYDMDNLRITTPKRNIDIERGQ

dCE9
SKRNKPGKATGKGKPVGDKWLDDAGKDSGAPIPDRIADKLRDKEFKSFDD
FRKAVWEEVSKDPELSKNLNPSNKSSVSKGYSPFTPKNQQVGGRKVYELN
HDKPISQGGEVYDMDNIRVTTPKRNIDIERGK

TrapA
MERYESLFAQLKERKEGAFVPFVTLGDPGIEQSLKIIDTLIEAGADALELGIPFS
DPLADGPTIQNATLRAFAAGVTPAQCFEMLALIRQKHPTIPIGLLMYANLVFNK
GIDEFYAQCEKVGVDSVLVADVPVEESAPFRQAALRHNVAPIFICPPNADDDLL
RQIASYGRGYTYLLSRAGVTGAENRAALPLNHLVAKLKEYNAAPPLQGFGIS
APDQVKAAIDAGAAGAISGSAIVKIIEQHINEPEKMLAALKVFVQPMKAATRS

TrapB
TTLLNPYFGEFGGMYVPQILMPALRQLEEAFVSAQKDPEFQAQFNDLLKNYA
GRPTALTKCQNITAGTNTTLYLKREDLLHGGAHKTNQVLGQALLAKRMGKTE
IIAETGAGQHGVASALASALLGLKCRIYMGAKDVERQSPNVFRMRLMGAEVI
PVHSGSATLKDACNEALRDWSGSYETAHYMLGTAAGPHPYPTIVREFQRMIG
EETKAQILEREGRLPDAVIACVGGGSNAIGMFADFINETNVGLIGVEPGGHGIE
TGEHGAPLKHGRVGIYFGMKAPMMQTEDGQIEESYSISAGLDFPSVGPQHAY
LNSTGRADYVSITDDEALEAFKTLCLHEGIIPALESSHALAHALKMMRENPDK
EQLLVVNLSGRGDKDIFTVHDILKARGEI
